# Excellent Adherence to Antiretrovirals in HIV+ Zambian Children Is Compromised by Disrupted Routine, HIV Nondisclosure, and Paradoxical Income Effects

**DOI:** 10.1371/journal.pone.0018505

**Published:** 2011-04-21

**Authors:** Jessica E. Haberer, Adrian Cook, A. Sarah Walker, Marjorie Ngambi, Alex Ferrier, Veronica Mulenga, Cissy Kityo, Margaret Thomason, Desiree Kabamba, Chifumbe Chintu, Diana M. Gibb, David R. Bangsberg

**Affiliations:** 1 Massachusetts General Hospital Center for Global Health, Boston, Massachusetts, United States of America; 2 Medical Research Council Clinical Trials Unit, London, United Kingdom; 3 University Teaching Hospital, Lusaka, Zambia; 4 Joint Clinical Research Centre, Kampala, Uganda; Karolinska Institutet, Sweden

## Abstract

**Introduction:**

A better understanding of pediatric antiretroviral therapy (ART) adherence in sub-Saharan Africa is necessary to develop interventions to sustain high levels of adherence.

**Methodology/Principal Findings:**

Adherence among 96 HIV-infected Zambian children (median age 6, interquartile range [IQR] 2,9) initiating fixed-dose combination ART was measured prospectively (median 23 months; IQR 20,26) with caregiver report, clinic and unannounced home-based pill counts, and medication event monitoring systems (MEMS). HIV-1 RNA was determined at 48 weeks. Child and caregiver characteristics, socio-demographic status, and treatment-related factors were assessed as predictors of adherence. Median adherence was 97.4% (IQR 96.1,98.4%) by visual analog scale, 94.8% (IQR 86,100%) by caregiver-reported last missed dose, 96.9% (IQR 94.5,98.2%) by clinic pill count, 93.4% (IQR 90.2,96.7%) by unannounced home-based pill count, and 94.8% (IQR 87.8,97.7%) by MEMS. At 48 weeks, 72.6% of children had HIV-1 RNA <50 copies/ml. Agreement among adherence measures was poor; only MEMS was significantly associated with viral suppression (p = 0.013). Predictors of poor adherence included changing residence, school attendance, lack of HIV disclosure to children aged nine to 15 years, and increasing household income.

**Conclusions/Significance:**

Adherence among children taking fixed-dose combination ART in sub-Saharan Africa is high and sustained over two years. However, certain groups are at risk for treatment failure, including children with disrupted routines, no knowledge of their HIV diagnosis among older children, and relatively high household income, possibly reflecting greater social support in the setting of greater poverty.

## Introduction

Over two million children under 15 years of age live with HIV/AIDS, 90% of whom reside in sub-Saharan Africa.[1] Efforts to provide children with antiretroviral therapy (ART) are increasing. By the end of 2008, 275,000 children had received ART, representing 38% of children estimated to need it.[2]

ART use in HIV-infected children leads to reduced plasma HIV RNA levels, increased CD4 cell counts, decreased incidence of opportunistic infections, improved growth and development, and decreased morbidity and mortality.[3], [4] High levels of sustained adherence, however, are needed to achieve these benefits.[5], [6]

A review of ART adherence in low and middle-income countries found a range in adherence level estimates from 49% to 100% with 76% of articles reporting >75% adherence.[7] The factors associated with adherence behavior among children are poorly understood and likely different from adults. A better understanding of the determinants of adherence is necessary to improve adherence and treatment outcomes. This need is particularly important in resource-constrained settings with limited treatment options.

This study presents adherence data from 96 Zambian children who were followed prospectively for up to two years. The two goals of the study were to 1) conduct a comparative evaluation of several widely used adherence measures; and 2) identify factors associated with adherence to fixed-dose combination (FDC) tablets in a resource-limited setting.

## Methods

### Ethical statement

Ethical approval for the parent trial CHAPAS-1 (Children with HIV in Africa – Pharmacokinetics and Adherence of Simple antiretroviral regimens, ISRCTN 31084535) and the adherence study was given by the University of Zambia and the University College, London. Informed, written consent was obtained from the parent or guardian of all participants. If he or she could not write, a witnessed thumbprint was accepted.

### Study population–The parent trial CHAPAS-1

The study population was drawn from the CHAPAS-1 trial, which was a randomized study of nevirapine (NVP) dose escalation among HIV-infected children initiating ART.[8] All children were treated at the University Teaching Hospital in Lusaka, Zambia. Children randomized to initiate NVP at full dose used FDC tablets of stavudine (d4T), lamivudine (3TC), and NVP (Triomune Baby/Junior) twice daily. Children randomized to escalate their dose of NVP used Triomune Baby/Junior once daily for 14 days, together with an FDC of d4T and 3TC (Lamivir-S) once daily. After 14 days Lamivir-S was stopped and children continued on twice daily Triomune Baby/Junior. FDCs were dissolvable, scored mini-tablets administered according to World Health Organization weight bands.[9] The CHAPAS-1 trial ran from February 2006 to December 2008, enrolling 211 children.

### Adherence measures in the parent trial

Adherence was measured at four-weekly clinic visits with pill counts, caregiver report of the last missed dose of ART (i.e. caregivers were asked “When did your child last miss any ART: within the last week, 1–2 weeks, 2–4 weeks, 1–3 months, nothing in 3 months?”), and a visual analog scale (VAS), where caregivers indicated the child’s adherence on a line marked with “none given” and “all given” at the ends and “half given” at the mid-point. Socio-economic data was collected at baseline and after six months, one year, and two years. Viral load was determined at 48-weeks from samples separated and frozen on site and processed at the Joint Clinical Research Centre Laboratory in Kampala, Uganda (Roche Amplicor Monitor version1.5 ultrasensitive assay).

### Adherence measures in the sub-study

The adherence sub-study ran from May 2006 until December 2008. It included two additional measures: 1) electronic monitoring with MEMS (Medication Event Monitoring System, Aardex, Switzerland) caps; and 2) unannounced monthly home visits for further pill counts. MEMS data was downloaded at each four-weekly clinic visit. Children stopped using MEMS caps when tuberculosis treatment required substitution of efavirenz for nevirapine and hence separate drugs rather than the FDC tablets. Drugs were dispensed four-weekly, and no pharmacy stock outs occurred during the trial.

### Analysis methods

To analyze agreement among methods, the four-weekly measurements of each child were averaged over the total follow-up to produce a summary adherence measure. For analysis of factors associated with MEMS adherence, data was aggregated into 12-week periods for each child, reducing random fluctuation and allowing a more prolonged effect of cofactors. The last MEMS period for most children covered <12 weeks.

Clinic pill counts were adjusted for children returning off-schedule. For each four-week period, MEMS data was used to calculate ‘taking adherence’; two or more bottle openings represented an adherent day, and days with a single opening counted as half-adherent. Adherence data from the first four weeks of treatment was not analyzed since dose escalation used two different tablets. For each MEMS cap, the last four-week time period was excluded if data were missing at the end of the period, because this finding commonly signalled cap failure or device non-use. For the last reported missed dose, each four-week period was classified as adherent or non-adherent, with an adherent period defined as no missed dose reported. Each child’s summary adherence measure was the percentage of adherent periods (e.g. no missed doses reported at nine of ten follow-up visits equals 90% adherence).

Agreement between adherence methods was assessed by Kappa statistics and Bland/Altman plots.[10] To calculate Kappa statistics, children were categorized as having adherence <80% versus ≥80% and <95% versus ≥95% for each adherence method. Bland/Altman plots illustrate agreement between two methods by plotting the difference between each pair of measurements against their mean.

Associations were investigated between viral load and average adherence at 48 weeks for each measure. Undetectable viral load was defined as <50 copies/ml, as adherence over the preceding 48 weeks was categorized as <80% versus ≥80% and <95% versus ≥95%. Where Fisher’s exact tests found significant associations, evidence for non-linearity in the relationship between viral load failure and adherence was explored via logistic regression using cubic splines for adherence.[11]

To identify predictors of adherence, random-effects regressions were used to model repeated measures from each child with time-updated factors. Poisson regressions were used to model the number of non-adherent days in each 12-week period with normally distributed between-subject error on the log-linear scale. Factors investigated included child and caregiver characteristics, socio-demographic status, and treatment-related factors. Univariate models were fitted separately to each factor, and those with significant univariate associations (p≤0.10) were used in a backward elimination (exit p>0.05) to create a multivariate model. Evidence of non-linear associations was investigated using cubic splines, with non-linearity represented by piece wise linear factors in final models.[11]

All analyses were performed using Stata statistical software (release 11, StataCorp, USA).

## Results

The first 107 consenting children were enrolled into the adherence sub-study. These children were statistically similar to other children in the parent study in age, sex, and disease stage (p>0.05). Nine children in the adherence sub-study died or were lost to follow-up shortly after randomisation, and two others had no MEMS data (one switched early to efavirenz, one stopped using MEMS for unknown reasons), leaving 96 participants for analysis. The median age at ART initiation was 6 (IQR 2,9) years, and 53 (55%) participants were male (Table 1). Many participants had advanced HIV disease at enrolment and were severely wasted and stunted. The primary caregiver was the mother for 68% of children. Sixty-six percent of children had more than one caregiver, with caregiver defined as someone who gives the child medication. In this primarily urban population, family size and dwellings were generally small, but 65% had electricity. Levels of poverty were high; median monthly household income was 398,000 Kwacha (US$79) and half of households spent ≥25% of income on food.

**Table 1:** Characteristics at ART initiation.

		N^a^	(%)^a^
Characteristic		96	(100)
Child			
Sex	Male	53	(55)
Age, years	Median (IQR)	6	(2,9)
WHO stage	3	60	(63)
	4	36	(37)
CD4%	≥15%	34	(35)
	<15%, ≥5%	53	(55)
	<5%	9	(9)
CD4 in children >5 years	Median (IQR)	379	(267,692)
Weight-for-age^b^	Median (IQR)	-3.2	(-4.2, -2.1)
	Z ≤-2SD	73	(76)
Height-for-age^b^	Median (IQR)	-3.1	(-4.1, -2.2)
	Z ≤-2SD	74	(77)
Attending school^c^	Yes	44	(46)
Knows their HIV status (9–15 years)^c^	Yes	2	(2)
Caregiver			
Primary caregiver	Mother	65	(68)
	Aunt	13	(14)
	Grandmother	10	(10)
	Father	4	(4)
	Other	4	(4)
No. of caregivers	1	32	(33)
	2	56	(58)
	3	8	(8)
Household^c^			
No. of other children	0	17	(18)
	1	20	(21)
	2	29	(31)
	≥3	29	(31)
Other household member on ART	Yes	18	(19)
No. of rooms	1–2	46	(48)
	>3	49	(52)
Electricity	Yes	61	(64)
Domestic tap	Yes	41	(43)
Monthly income (in 1000 Kwacha)	Median (IQR)	398	(250,700)
Main income source	Market worker	49	(52)
	Driver	5	(5)
	Public servant	13	(14)
	Farmer	6	(6)
	Security/police	4	(4)
	Other	18	(19)
Food as percent expenditure	Median (IQR)	27	(20,47)

a. Other statistics are indicated in the second column.

b. UK 1990 growth reference; WHO 2007 reference only available to 10 years. For children to age 10, the average UK weight Z-score was 0.5 lower than the WHO reference and the average UK height Z-score was 0.1 higher than WHO reference.

c. Missing data for one child.

Total follow-up was 164.4 person years (pyrs) from 2134 clinic visits, with a median of 23 follow-up visits per child (IQR 20,26: range 1,31). Follow-up was shorter for MEMS caps (113.5 pyrs), as 23 children had caps replaced during the study because of loss or failure. Additionally, MEMS follow-up ended for 30 children in early 2008 when the bottle changed and was incompatible with MEMS caps. Follow-up from home visits was also shorter because they were often prevented by transport problems and the visit occurred approximately three weeks into each month; 1275 total visits took place covering 71.7 pyrs. Three children included in the analysis died and five others were lost to follow-up. The median follow-up among these eight children was 14 weeks (IQR 8,36).

Overall adherence to twice daily FDC was high (Table 2). VAS and clinic pill counts indicated median adherence of 97.4% (IQR 96.1,98.4%) and 96.9% (IQR 94.5,98.2%), respectively. These clinic-based measures also indicated relatively few children with low adherence. Sixteen percent had adherence <95% by VAS, and 31% had adherence <95% by clinic-based pill count. No children had adherence <80% by either method. Caregivers were asked the last missed dose question a median of 23 times (IQR 20,26), and the median number of times missed doses were reported was 1 (IQR 0,3), giving a median adherence of 94.8% (IQR 86,100%). Over 25% of caregivers reported no missed doses ever. Median adherence from unannounced home-visit pill counts and MEMS data was also high at 93.4% (IQR 90.2,96.7%) and 94.8% (IQR 87.8,97.7%), respectively. These methods, however, also indicated higher levels of incomplete adherence compared to clinic-based measures. Unannounced home-visit pill counts indicated adherence <95% and <80% in 62% and 10% of children, while MEMS data indicated adherence <95% and <80% in 51% and 13% of children, respectively. The timing of MEMS events was well distributed in that two openings were at least eight hours apart for 96% of adherent days.

**Table 2:** Follow-up, summary of adherence measures and agreement between methods.

				Visual Analogue	Last missed dose
		Pill count			
	MEMS	Home visit	Clinic visit	Scale (VAS)	question
Follow-up, by method					
Total follow-up (pyrs)	113.5	71.7	163.2	163.3	163.3
Number of children	96	96	96	96	96
Median (IQR) visits/child	15 (10,20)	15 (10,18)	23 (20,26)	23 (20,26)	23 (20,26)
Summary of adherence, by method					
Adherence (median)	94.8%	93.4%	96.9%	97.4%	94.8%^a^
IQR	87.8,97.7	90.2,96.7	94.5,98.2	96.1,98.4	86,0,100
Range	31.3,100	67.5,100	83.4,100	87.5,100	64.3,100
Adherence <95%, n (%)	49 (51.0)	59 (61.5)	30 (31.2)	15 (15.6)	48 (50.0)
Adherence <80%, n (%)	12 (12.5)	10 (10.4)	0	0	15 (15.6)
Agreement between methods, Kappa statistic (95% CI)					
	MEMS	0.42 (0.26,0.58)	0.19 (0.04,0.35)	0.05 (0.0,0.18)	0.31^b^ (0.16,0.46)
	Home visit		0.36 (0.20,0.53)	0.18 (0.05,0.30)	0.24 (0.08,0.39)
	Clinic visit			0.52 (0.34,0.70)	0.15 (0.0,0.30)
	VAS				0.11 (0.0,0.23)

a. Percentage of follow-up visits with no missed dose reported during previous month.

b. Agreement between the last missed dose question and other methods should be interpreted separately (e.g. 90% adherent for the last missed dose means that no treatment was missed in nine periods out of ten, but does not indicate the level of non-adherence in those periods).

Agreement between methods was poor with low Kappa statistic values (Table 2). Unannounced home-based pill counts had higher agreement with MEMS data (Kappa = 0.42; 95%CI 0.26,0.58) than either clinic-based measure. The relatively good agreement between VAS and clinic pill counts (Kappa = 0.52; 95%CI 0.34,0.70) resulted from 70% of children having adherence >95% with both methods. The highest agreement with the last missed dose question was observed with MEMS data (Kappa = 0.31; 95%CI 0.16,0.46), while the lowest agreement was with the VAS (Kappa = 0.11; 95%CI 0.0,0.23). The last missed dose question does not measure how much ART was missed; rather, it indicates that not all doses were taken and should be interpreted separately. Disagreement between methods appeared to be greater in some children compared to others. Eight children accounted for over half of the 107 clinic visits in which MEMS data indicated adherence <80% and clinic pill counts simultaneously indicated adherence >95%.

**Figure 1:**
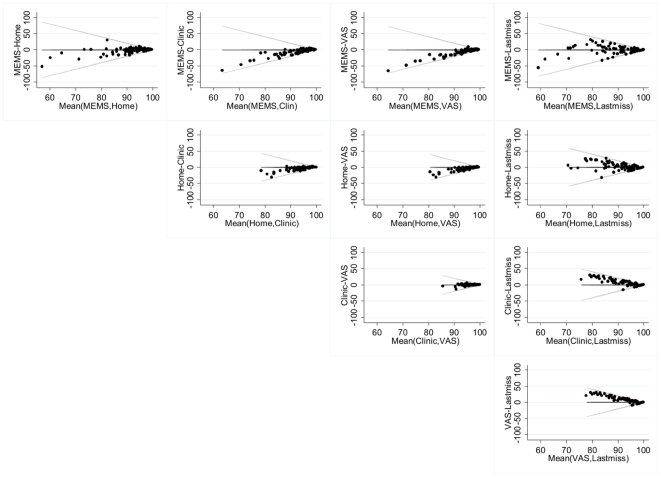
Agreement between measures (as shown by the difference between methods versus mean adherence value for each child). The Bland-Altman plots in this figure show pair wise agreement between adherence methods. Each plot indicates the difference between two methods on the vertical axis against the mean of the same methods on the horizontal axis. Data points above the zero line occur when the first method shows higher adherence than the second. On the horizontal axis, data points to the right indicate high adherence from both methods, in which case the maximum possible difference between them is shown by the angled lines.

Agreement between methods is further illustrated by Bland/Altman plots (Figure 1), which show that, when compared to MEMS data or home-based pill counts, clinic pill counts and VAS consistently estimated higher adherence with almost no data above the zero line. Better agreement was shown between MEMS and home-based pill counts, but again a tendency for pill counts to give higher estimates was observed. The plot of clinic pill counts against VAS shows the very limited data range. For the last missed dose question, the highest agreement was seen with MEMS data, while other clinic-based measures consistently gave higher estimates of adherence.

Viral load was measured at 48 weeks in 73 of 96 children (76%) and was <50 copies/ml in 53 of 73 (73%). Data for 23 children were unavailable because of death (N = 2), loss to follow-up (N = 4), or inadequate samples (N = 17). Viral load was significantly associated with poor MEMS adherence (p = 0.013); five of seven children (71%) with adherence <80% had detectable viral load (Table 3). No other adherence measure had a significant association with viral load. Odds of suppression increased linearly over the range of MEMS adherence observed with no evidence of a specific threshold (non-linearity p = 0.47).

**Table 3:** Association between adherence measures and viral load.

	Viral load			
	<50 copies/ml	≥50 copies/ml		
	N (%)	N (%)		
	53 (73%)	20 (27%)	N	P
MEMS adherence				
≥95%	35 (81%)	8 (19%)	43	0.013
≥80%,<95%	15 (68%)	7 (32%)	22	
<80%	2 (29%)	5 (71%)	7	
Home visit pill count				
≥95%	21 (70%)	9 (30%)	30	0.91
≥80%,<95%	29 (74%)	10 (26%)	39	
<80%	3 (75%)	1 (25%)	4	
Clinic based pill count				
≥95%	27 (67%)	13 (33%)	40	0.31
≥80%,<95%	26 (79%)	7 (21%)	33	
<80%	0	0	0	
Visual analogue scale				
≥95%	32 (70%)	14 (30%)	46	0.59
≥80%,<95%	21 (78%)	6 (22%)	27	
<80%	0	0	0	
Last missed dose				
≥95%	24 (77%)	7 (23%)	31	0.70
≥80%,<95%	19 (68%)	9 (32%)	28	
<80%	10 (71%)	4 (29%)	14	

MEMS follow-up ranged between one and ten 12-week periods per child (median = 5; IQR 4,7). The outcome for each time period was the number of days of missed ART. Socio-economic data was not available for ten children until the six-month time period. A total of 509 time periods were analyzed from 95 children. The median number of missed ART days in the 12-week periods was 4 (IQR 1,10).

Factors that were not significant in univariate analyses were early or late entry into the trial, change of primary caregiver, total number of changes in caregiver, child gives himself or herself ART, socioeconomic index (a first principal component based on household possessions), and change of dose. Factors significant in univariate analyses but not selected in the final multivariate model were the number of tablets daily, differing morning and evening ART doses, other household member on ART, main occupational source of household income, and percentage of household income spent on food.

The factors included in the multivariate model are shown in Table 4. Several child characteristics were found to significantly affect MEMS adherence. A complex relationship was observed between age and sex. Missed ART days among boys decreased by 44% annually up to five years, increased by 56% between five and ten years, and decreased by 25% above ten years. Missed ART days among girls decreased by 27% annually up to five years, continued to decrease by 10% between five and ten years, and increased by 31% above ten years. Across the age range, missed ART days were not consistently higher in boys or girls, but were significantly higher in girls at age five (IRR 3.50; 95%CI 1.20,10.21) and significantly lower in girls at age ten (IRR 0.23; 95%CI 0.08,0.65).

**Table 4:** Predictors of the number of non-adherent days per quarter.

		Univariate model			Multivariate model		
Variable		IRR	95%CI	P	IRR	95%CI	P
Child related							
Sex							
Female		1.35	0.74,2.47	0.323			
Age							
<5yrs (per year)		1.10	0.96,1.26	<0.001			
5-<10yrs		1.47	1.28,1.69				
≥10yrs		1.15	0.99,1.34				
Age and sex							<0.001^a^
boy <5yrs (per year older)					0.56	0.44,0.72	
boy 5-<10yrs					1.56	1.29,1.89	
boy ≥10yrs					0.75	0.60,0.94	
girl <5yrs					0.73	0.56,0.94	
girl 5-<10yrs					0.90	0.73,1.11	
girl ≥10yrs					1.31	1.00,1.72	
girl : boy at age 5					3.50	1.20,10.21	<0.001^b^
girl : boy at age 10					0.23	0.08,0.65	
CD4% (5% higher)		1.10	1.07,1.14	<0.001	1.05	1.01,1.09	0.016
Weight-for-age Z (unit higher)		1.46	1.34,1.58	<0.001	1.34	1.20,1.50	<0.001
Attends school		2.94	2.40,3.61	<0.001	1.46	1.14,1.88	0.003
Knows their HIV status		0.65	0.50,0.83	0.001	0.62	0.47,0.81	0.001
Caregiver related							
Primary caregiver	Mother	1.00		<0.001	1.00		<0.001
	Aunt	2.19	1.61,2.96		2.57	1.84,3.58	
	Grandparent	1.29	0.91,1.83		1.34	0.91,1.97	
	Other	1.41	1.09,1.82		1.35	1.01,1.79	
No. of caregivers	1	1.00		0.002	1.00		<0.001
	2	0.79	0.70,0.89		0.77	0.68,0.88	
	≥3	0.90	0.74,1.10		0.82	0.66,1.03	
Caregiver knowledge of ART		1.37	1.26,1.49	<0.001	1.25	1.13,1.38	<0.001
Household related							
Change of address		1.33	1.08,1.65	0.008	1.57	1.25,1.97	<0.001
No. of other children (per child)		1.42	1.31,1.53	<0.001	1.26	1.15,1.39	<0.001
Income (per 100,000 Kwacha)							
up to 800,000 per month		1.12	1.07,1.17	<0.001	1.08	1.03,1.14	0.003
above 800,000 per month		1.01	0.99,1.03		0.99	0.96,1.01	
Other							
Months in study	0–3	1.00		<0.001	1.00		<0.001
	4–6	1.08	0.95,1.23		0.94	0.82,1.08	
	7–9	0.93	0.81,1.06		0.77	0.66,0.91	
	10–12	1.12	0.97,1.28		0.74	0.62,0.88	
	13–15	1.35	1.17,1.57		0.82	0.66,1.01	
	16–18	1.43	1.22,1.67		0.90	0.71,1.14	
	≥19	1.90	1.63,2.22		1.18	0.91,1.52	

IRR – Incidence rate ratio, all factors time updated except for sex.

a. Overall p-value for age and sex.

b. P-value for interaction between age and sex.

The number of missed ART days increased by an average of 34% with a unit increase in weight-for-age Z-score (p<0.001) and by 5% for each increase in CD4% of 5% (p = 0.016). A child’s knowledge of his/her HIV status and attendance at school were strongly associated with age, but remained independent predictors with adjustment for age. Two children knew their HIV status at baseline, and 24 others aged nine to 15 years learned it during the trial. The average number of missed ART days was 46% higher among those attending school (p = 0.003), but 38% lower among children knowing their status compared to children who did not know (p = 0.001).

A significant improvement in adherence was also observed after six months (p<0.001), which remained when analysis was restricted to children with ≥18 months follow-up.

Caregiver and household characteristics were also significant covariates. The highest adherence was observed where the child’s mother was the primary caregiver (p<0.001), and improved by 23% if the child had multiple caregivers, but the effect of two caregivers was similar to three or more (p<0.001). Adherence was 25% worse among children whose caregivers reported giving ART because they knew why their child needed it versus caregivers who did not know why (p<0.001). Missed ART days increased by 57% after changing address (p<0.001), and by 26% for each other child in the household (p<0.001). Missed ART days increased by 8% per 100,000 Kwacha (US$20) income up to 800,000 Kwacha (US$160) per month, and did not increase further at higher incomes (p = 0.001). Total household income was not directly adjusted for household size, although the number of caregivers and other children was included in the model.

## Discussion

This study is the first in-depth analysis of ART adherence among HIV-infected children with nearly two years of follow-up in sub-Saharan Africa. The high levels of overall adherence are encouraging, as they are likely adequate for viral suppression with the non-nucleoside reverse transcriptase-based regimens typically available for children in developing settings.[9], [12] Some of the high adherence may have been facilitated by the convenience of taking FDC tablets.[13] In the absence of FDC, pediatric regimens are commonly complex, involving multiple medications and syrups that change frequently according to child development and drug availability.[14] The lack of a comparison group, however, limits this conclusion. The longitudinal improvement in adherence is also very positive and contrasts with a recent study of mother-child dyads in Kampala showing decreasing trends in adherence.[15] The findings that 27% of children had detectable viral load at 48 weeks and 18% of children had <80% adherence by MEMS, however, indicate that subpopulations are at risk for treatment failure.

The negative effect of change in residence suggests the child’s adherence may reflect household instability and some aspects of routine may be important, although no independent effect was seen for change of caregiver or change of dose. The negative impact of school attendance may also reflect disruption of routine, as children may board at the school, take breaks between semesters, and may have difficulty taking their medicine confidentially.

Effects of HIV disclosure to children on adherence are complex,[16] and little is known in sub-Saharan Africa. Two qualitative studies identified limited communication between caregivers and children, as well as the need for better support throughout the disclosure process.[17], [18] The improved adherence with HIV disclosure to the child suggests that full understanding of HIV medications is important in these children. Paradoxically, adherence was worse when caregivers had good knowledge of why their child needed ART. This association may be explained by the finding that improved caregiver knowledge was significantly associated with another household member being on ART (OR = 1.64, p = 0.021), reflecting households with greater HIV burden. Further exploration of this important issue is needed.

The adherence pattern seen with household incomes suggests that families with fewer financial resources have better adherence than those with higher incomes. Better adherence in the setting of low resources may be explained by enhanced social support among people living in extreme poverty, which has been proposed as an explanation for good adherence among adults in sub-Saharan Africa.[19] According to this theory, individuals taking ART overcome economic obstacles to adherence through the assistance of family and other supporters (e.g. borrowing transport funds, sharing resources during the time needed to attend clinic). In exchange, these supporters expect adherence, creating a responsibility for patients to adhere. Indeed, the worsened adherence seen with increased numbers of children in the household may reflect economic and logistical challenges to sustained HIV care. This finding should not be misinterpreted to suggest that poverty is a means to improve adherence. Rather, social support differs by socio-economic level, and greater reliance on close social networks among people living in poverty may enhance adherence. The worsened adherence with increased weight-for-age Z-score and CD4% may reflect decreased motivation to adhere as children regain health and have fewer HIV-related symptoms.[20]

The influence of age and sex on adherence was notable. Older children are often reported to have lower adherence than both younger children and adults.[14] Relatively few studies on this topic have been published from sub-Saharan Africa; however, one from South Africa found lower adherence in adolescents compared to adults,[21] while another in Uganda found no difference in adherence by age or sex.[22] The authors are unaware of any studies examining the interaction of age and sex on adherence. The finding of worsening adherence for girls and improving adherence for boys over ten years could suggest a cultural bias toward supporting males. Alternatively, the number of children at this age is relatively small and this finding could be due to chance.

Although this study did not assess interventions to improve adherence, the findings suggest several potentially modifiable factors, including leveraging social support to overcome economic barriers to care, stabilizing routine possibly through additional caregiver support, and/or disclosing HIV to older children. Additionally, unexplained between-child variation remained in the final regression model, suggesting the presence of other unknown reasons why some children have consistently better adherence than others.

This study used multiple adherence measures, as no gold standard exists and each method has advantages and disadvantages. Subjectively reported adherence is easy to collect, but often felt to overestimate adherence.[23] Clinic pill counts are also relatively easy to perform and objective; however, participants may manipulate pills to appear more adherent,[24] which may explain the clustering of discordant MEMS and clinic pill count data within certain children. Home-based pill counts are performed unannounced, such that participants have little opportunity for pill manipulation, but they are resource-intensive.[25] MEMS is generally accepted as the industry standard for adherence measurement,[26] although they are expensive and preclude the use of pill box organizers.[27] Moreover, participants may open the cap without removing pills, remove multiple pills at a time, or simply not use it.[28]

As expected, median adherence in this study was higher for self-reported adherence and clinic pill counts compared to unannounced home-based pill counts and MEMS. MEMS was selected as the best measure in this study population because it had the least evidence of higher estimates compared to other measures and had the only significant association with viral load. Moreover, more precise measures by definition have greater ability to distinguish among different levels of adherence compared to less precise measures. MEMS gave the widest distribution of adherence, suggesting it was more precise than the other measures. The MEMS data also showed distinct morning and evening peaks, which may reflect true pill taking behavior.

Caregiver-report of last missed ART dose had a relatively high agreement with MEMS. These findings suggest that certain self-report questions may perform better than others. Indeed, various recall periods and question formats have been shown to correlate differently with MEMS.[29] Given the overall high adherence in this study, a missed dose may have been fairly easy to recall and more sensitive in detecting incomplete adherence than recalling the number or percentage of doses missed.[30]

This study had several limitations. First, only MEMS data was used to assess for predictors of adherence. Second, the study population was drawn from a clinical trial and the children may have been more motivated to adhere than children outside the research setting. Additionally, approximately 10% of participants did not complete the study, and their adherence behavior may have differed from children remaining in the study. Finally, the process of measuring adherence can improve adherence,[31] but the effect is usually temporary and affects a minority of individuals.[32]

In conclusion, this study provides encouraging data about high, sustained long-term adherence among children taking fixed-dose combination ART in sub-Saharan Africa. Multiple measures of adherence were used to estimate adherence behavior, and all suggest adherence is in the range needed for treatment success. That said, subgroups at risk were identified, including children with disrupted routine, no knowledge of their HIV infection, and relatively high incomes. Future research should focus on developing interventions to support such children.
